# Systematic review and meta-analysis of the prognostic significance of microRNAs related to metastatic and EMT process among prostate cancer patients

**DOI:** 10.1186/s12967-020-02644-x

**Published:** 2021-01-07

**Authors:** Martyna Parol, Arkadiusz Gzil, Magdalena Bodnar, Dariusz Grzanka

**Affiliations:** Department of Clinical Pathomorphology, Collegium Medicum in Bydgoszcz, Nicolaus Copernicus University in Torun, 9 Curie-Sklodowskiej Street, 85-094 Bydgoszcz, Poland

**Keywords:** Epithelial–mesenchymal transition, EMT, Metastases, microRNA, miRNA, Prostate cancer, Meta-analysis

## Abstract

The ability of tumor cells to spread from their origin place and form secondary tumor foci is determined by the epithelial–mesenchymal transition process. In epithelial tumors such as prostate cancer (PCa), the loss of intercellular interactions can be observed as a change in expression of polarity proteins. Epithelial cells acquire ability to migrate, what leads to the formation of distal metastases. In recent years, the interest in miRNA molecules as potential future treatment options has increased. In tumor microenvironment, miRNAs have the ability to regulate signal transduction pathways, where they can act as suppressors or oncogenes. MiRNAs are secreted by cancer cells, and the changes in their expression levels are closely related to a cancer progression, including epithelial–mesenchymal transition. These molecules offer new diagnostic and therapeutic possibilities. Therapeutics which make use of synthesized RNA fragments and mimic or block miRNAs affected in PCa, may lead to inhibition of tumor progression and even disease re-emission. Based on appropriate qualification criteria, we conducted a selection process to identify scientific articles describing miRNAs and their relation to epithelial–mesenchymal transition in PCa patients. The studies were published in English on Pubmed, Scopus and the Web of Science before August 08, 2019. Hazard ratios (HRs) and 95% confidence intervals (CI) as well as total Gleason score were used to assess the concordance between miRNAs and presence of metastases. A total of 13 studies were included in our meta-analysis, representing 1608 PCa patients and 15 miRNA molecules. Our study clarifies a relationship between the clinicopathological features of PCa and the aberrant expression of several miRNA as well as the complex mechanism of miRNA molecules involvement in the induction and promotion of the metastatic mechanism in PCa.

## Introduction

Prostate cancer (PCa) is the second most common diagnosed cancer and is considered the fifth leading cause of cancer-related deaths among men worldwide [1]. According to American Cancer Society statistics, the PCa incidence rates between 2011 and 2015 amounted 109.2 per year and the PCa death rates between 2012 and 2016 reached 19.2, showing decrease tendency [2] with the peak incidence (237.5 per year) and death rates (39.2) in 1992 [3]. In general, PCa has outstanding survival rates [3]. Surveillance, Epidemiology, and End Results Program (SEER 18 2009–2015) data shows the relative 5-year survival rate of local and regional stage PCa is almost 100%, for all races [2, 3]. However, for late stage PCa, the relative 5-year survival rate is only 30.5% [2, 3] Moreover, it is estimated that about 30% of men with diagnosed PCa have bone metastases [4]. In the U.S., the estimations for PCa with bone metastases amount to 6.5% (47,607 PCa with bone metastases in 735,812 of all PCa cases) of all PCa cases in 2004 [5]. Since 2007, the low-risk PCa incidence rate has decreased, however, the metastatic PCa incidence rate has notable increased [6].

The metastatic PCa involves multistep processes, including invasion, migration and survival of migrating cells, also location and proliferation in distant sites [7]. A critical point in metastases formation is attributed to the epithelial–mesenchymal transition (EMT) process [8]. EMT is a unique process by which tumor epithelial cells undergo molecular reprogramming and cytoskeleton remodeling through their transition to mesenchymal cell phenotype, The cells lose their adhesion capability and acquire the ability to move and to invade other tissues [9]. It is well known that the EMT process constitutes the early stage of metastases [10]. More specifically, the EMT transition is characterized by E-cadherin suppression associated with loss of cell adhesion properties and acquisition of mesenchymal markers, such as N-cadherin, Vimentin, Fibronectin, which enable cell mobility [11].

In the last decade, microRNAs have been shown to play an important role in managing single or numerous complex steps of metastases leading to tumor outgrowth by deregulation of target genes expression. MicroRNAs (miRNAs) are approximately 18–22 nucleotides long, single-stranded and non-coding regulatory RNAs, habitually aberrant in PCa [12, 13]. In normal tissue, microRNAs have the ability to regulate many biological processes such as cell proliferation, cell cycle arrest, aging and apoptosis. A number of studies have confirmed their presence and participation in metastases. They are responsible for controlling the expression level of prometastatic genes through base-paring with messenger RNAs (mRNAs) at the post-transcriptional level and lead to enhancement or suppression of the metastatic process.

Single miRNA has the ability to target multiple mRNA genes simultaneously. Many previous studies have revealed that above 50% of miRNA genes are situated in cancer-associated genomic regions and they form a central nodal points due to cancer progression [14]. They constitute crucial modulators in human cellular signaling pathways and are engaged in controlling gene expression of their targets. The abnormal expression level of miRNAs is heavily related to disease progression [15]. Owing to changes in expression levels, miRNAs have been classified as oncogenic molecules and tumor suppressors. Oncomirs (Onc) promote cancerogenesis through damping the mRNAs encoding tumor suppressor proteins at translational level (miR-301a, miR-409-3p, miR-210-3p, miR-543, miR-409-3p, miR-410-3p in PCa). While, tumor suppressors (TS) miRNAs are responsible for repression of mRNAs encoding onco-proteins at translational level (miR-573, miR-802, miR-3622a, miR-466, miR-203, miR-132/212, miR-141-3p in PCa).

MicroRNAs show strong promise as molecular biomarkers and treatment opportunity due to several aspects. Firstly, they manifest tissue-specific expression patterns in malignancies and exhibit a highly stable form in formalin-fixed tissues [16], as well as in blood, plasma and other bodily fluids [17, 18]. Circulating microRNAs are resistant to endogenous RNases activity, therefore their expression level can be determined in bodily fluids. Secondly, miRNA expression levels are varied in individual cancers, so they can specify different types of cancer. Thirdly, there has been observed a clear dissimilarity of miRNA regulation between different kinds of cancer. Fourthly, the efficiency and accuracy of miRNAs oriented to the target cells, make them attractive molecules in miRNA-based therapy. Fifthly, various microRNA profiles could be used for distinguish pathological and normal tissue as well as accurately recognize cancerous subtypes [19].

To the best of our knowledge there is no meta-analysis which describes the prognostic significance of miRNAs-related to the EMT process in PCa. In this study, we revealed the importance of fifteen deregulated miRNAs that constitute therapeutic potential and could be utilized as measurable indicators of PCa metastatic potential. To investigate their significance in EMT transition, we performed a meta-analysis, based on patient data extracted from scientific articles, where studies were conducted on formalin-fixed paraffin-embedded or fresh/snap-frozen tissues.

## Materials and methods

### Search strategy and eligibility criteria

The selected publications were identified by using up-to-date electronic databases, including PUBMED, Scopus and Web of Science online databases. The following keywords were used for the search: miRNA, EMT and prostate cancer. The inclusion criteria were as follows: (1) type of microRNA (miRNA was related to EMT process); (2) the articles were published between 2010 and 2019 year; (3) the study was conducted on formalin-fixed paraffin embedded tissue samples (FFPE) or fresh/snap-frozen prostate cancer tissues; (4) the study contained the following clinicopathological descriptions: TNM stages, according to TNM Classification of Malignant Tumors system (TNM) as well as Gleason score (GS) which were grouped by both low and high level of microRNAs expression. Exclusion criteria were as follows: (1) review articles; (2) studies based on PC cell lines; (3) non-clinical samples; (4) lacked sufficient data for estimating Hazard Ratios (HRs) and their 95% confidence intervals (CIs); (5) duplicate articles; (6) non-English paper; (7) case reports, letters, commentaries, conference abstract. The publication list was last updated on August 08, 2019.

### Quality assurance

Methodology quality evaluation of incorporated studies was performed by two reviewers, working independently with the Newcastle–Ottawa Scale (NOS) for case–control quality assessment [20]. The evaluation process was divided into three steps: selection with a maximum score of 4, comparability with a maximum score of 2 and exposure/outcome with a maximum score of 3. A total score of 6–9 points ensured the highest quality.

### Data extraction

The data extraction process was provided irrespectively two times by one author (MP) and was supervised by the senior reviewers in case of discrepancies (MB, DG). We collected the following items extracted from all included publications: (1) basic details: the title, first author’s last name, year of publication, country/continent of origin; (2) general details of the study: the name of microRNAs, sample size and kind of sample, microRNA detection method, size of both the low and high group of microRNAs expression level; (3) clinicopathological details: the TNM Classification of Malignant Tumors and Gleason score.

### Statistical analysis

All statistical analyses were completed with the PQStat package. *P* value < 0.05 was considered as statistically significant. Estimation of miRNAs expression in PCa patients and their prognostic effects was presented using forest plots. We used the fixed-effects model to calculate Relative Risks (RRs) and 95% CIs in all included studies. In general, a RR > 1 demonstrated a poor patient outcome in cases with higher miRNA expression. The Cochrane’s Q statistic and Inconsistency index (I^2^) test was used to assess of heterogeneities. An I^2^ ≥ 50% is considered as a high heterogeneity level. Funnel plots were used to visualize of publication bias.

## Results

### Study characteristics

Study selection process identified 13 articles, which were included in the meta-analysis. Our study worked with articles published between 2010 and 2019, focusing on data of following miRNA: miR-410-3p [21], miR-573 [22], miR-3622a [23], miR-301a [24], miR-466 [25], miR-503 [26], miR-203 [27], miR-210-3p [28], miR-802 [29], miR-543 [30], miR-132 and miR-212 [31], miR-409-3p and miR-409-5p [32] and miR-141-3p [33].

**Fig. 1:**
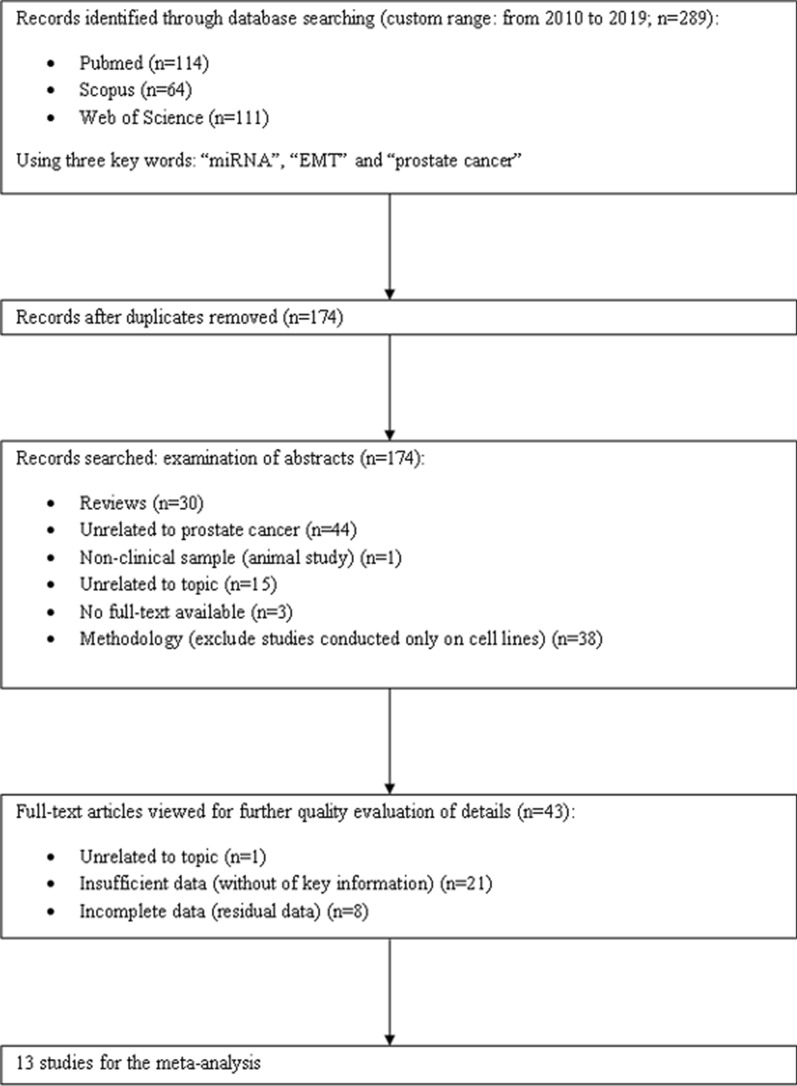
The flow diagram of meta-analysis

The total of 289 primary articles were searched in PubMed, Scopus and Web of Science databases. The flow chart diagram of the study selection process in our meta-analysis is presented below (Fig. 1). In the first step, we excluded 115 duplicated literatures. After examination of abstracts and full-text articles, we removed 161 publications and included 13 articles, finally. The main characteristics of involved studies was recapitulated in Table 1. The miRNAs in Table 1 were arranged in alphabetical order and some articles were divided into two parts because of more described miRNAs. The total group of the meta-analysis incorporated 1608 patients. The enrolled participants were divided into clinical and pathological stages, according to the TNM Classification of Malignant Tumors (TNM) standard. The participants of included studies mainly derived from China and the USA, but the dominant ethnicity was Chinese in approximately 60% of all studies. Detection of miRNAs deregulation was accomplished using real-time polymerase chain reaction (RT-PCR). The miRNAs fraction was isolated from fresh/snap-frozen or formalin-fixed paraffin embedded sections of tissue.

**Table 1:** The main characteristic of included studies

Author	Publication year	Country	miRNA type	General details of the study		
				Sample type	Sample size	Detection method
Bucay	2017	USA	miR-3622a	FFPE	138	RT-PCR
Colden	2017	USA	miR-466	FFPE	96	RT-PCR
Du	2017	China	miR-543	Fresh	42	RT-PCR
Fu	2016	China	miR-132	FFPE	57	RT-PCR
Fu	2016	China	miR-212	FFPE	57	RT-PCR
Huang	2017	China	miR-141-3p	Snap-frozen	141	RT-PCR
Jiang	2016	China	miR-503	FFPE	82	RT-PCR
Josson	2017	USA	miR-409-3p	Tissue microarray	61	RT-PCR
Josson	2017	USA	miR-409-5p	Tissue microarray	61	RT-PCR
Nam	2016	Canada	miR-301a	FFPE	585	RT-PCR
Ren	2017	China	miR-210-3p	Snap-frozen	149	RT-PCR
Saini	2010	USA	miR-203	FFPE	22	RT-PCR
Wang	2015	China	miR-573	FFPE	80	RT-PCR
Wang	2017	China	miR-802	Fresh	73	RT-PCR
Zhang	2018	China	miR-410-3p	Fresh	82	RT-PCR

### Associations between miRNAs and the clinicopathological features of prostate cancer

In the current meta-analysis, we checked the association between miRNAs and clinicopathological features of prostate cancer presented in Table 2. For these compilations the Relative Risk (RR) and corresponding 95% Confidence Intervals (95% CI) were calculated.

**Table 2:** Associations between miRNAs and clinicopathological features studied in current meta-analysis

Clinicopathological features	miRNAs	Figures
Presence of metastasis		
GM	miR-410-3p, miR-132, miR-212, miR-802, miR-141-3p, miR-210-3p, miR-301a, miR-503, miR-543	Figure 2a
LNM	miR-802, miR-301a, miR-503	Figure 2b
DM	miR-132, miR-212, miR-802, miR-141-3p, miR-210-3p	Figure 2c
BM	miR-141-3p, miR-210-3p	Figure 2d
Total Gleasone scor		
GS > 6	miR-410-3p, miR-132, miR-212, miR-203, miR-409-3p, miR-409-5p, miR-301a, miR-466, miR-503, miR-543, miR-573, miR-3622a	Figure 2e
GS > 7	miR-132, miR-212, miR-802, miR-141-3p, miR-210-3p, miR-203, miR-409-3p, miR-409-5p, miR-301a, miR-466, miR-503, miR-543, miR-573, miR-3622a	Figure 2f
pT stage according to TNM	miR-410-3p, miR-132, miR-212, miR-802, miR-203, miR-301a, miR-466, miR-503, miR-543, miR-573, miR-3622a	Figure 2g

*GM* general metastasis, *LNM* lymph node metastasis, *DM* distant metastasis, *BM* bone metastasis, *GS* Gleason score, *pT* stage-pathological tumor stage

### Association between miRNAs and the metastasis generally

**Fig. 2:**
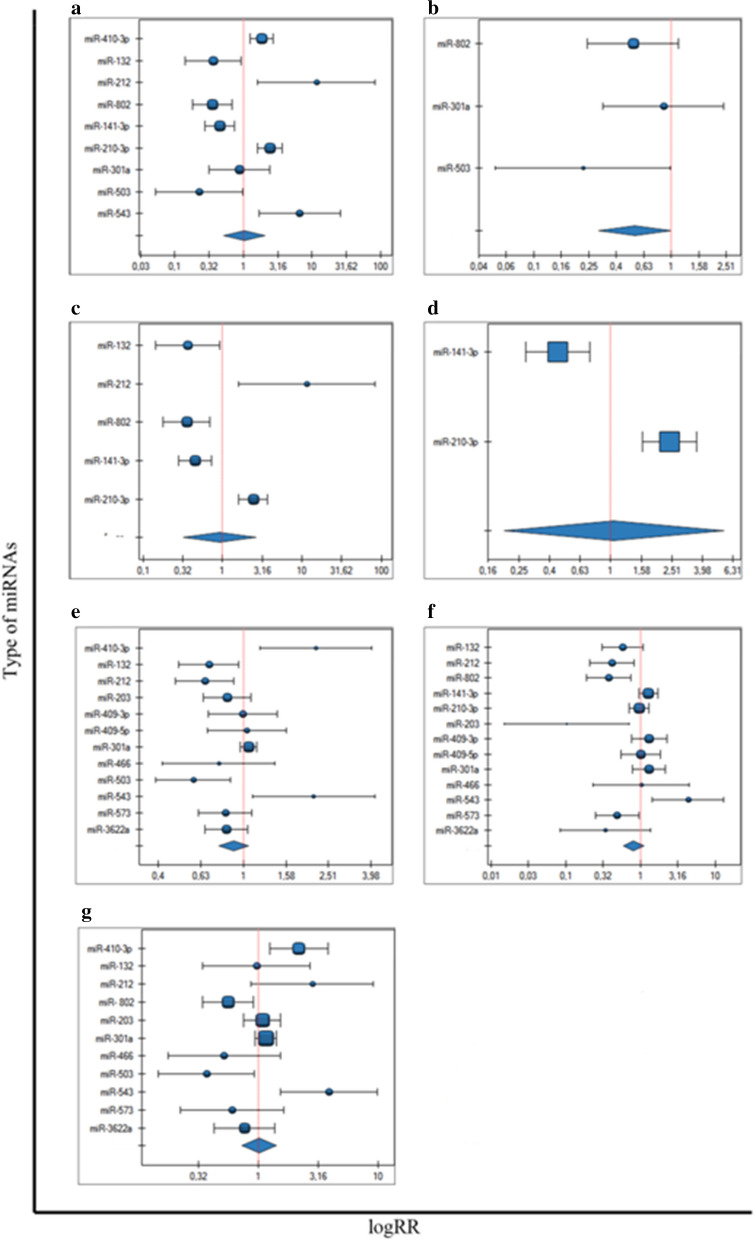
Forest plot analysis

A total of eight articles, involving ten miRNA molecules, were included to analysis of the association between miRNAs expression and the presence of metastasis. The total number of included patients was 881 individuals. The heterogeneity of this analysis was at high level (*p* < 0.000001, I^2^ = 87%). Patients with high expression of miR-410-3p, miR-212, miR-210-3p and miR-543 had significantly poorer prognosis compared to their low expression level respectively (RR = 1.85, 95% CI 1.25–2.74, *p* = 0.002; RR = 11.59, 95% CI 1.61–83.31, *p* = 0.014; RR = 2.43, 95% CI 1.61–3.67, *p* = 0.000; RR = 6.60, 95% CI RR = 1.68–25.95, *p* = 0.005) (Fig. 2a). Furthermore, low expression of other four miRNAs, miR-132, miR-802, miR-141-3p, miR-503 were predicted significantly to tumor progression, compared to high expression of them (RR = 0.36, 95% CI 0.14–0.92, *p* = 0.033; RR = 0.35, 95% CI 0.18–0.69, *p* = 0.002; RR = 0.45, 95% CI: 0.28–0.73, *p* = 0.001; RR = 0.23, 95% CI 0.05–0.99, *p* = 0.05). In the one analysis with miR-301a no statistically significant association was noticed (Fig. 2a). The clinicopathological details of the group, concerning information about metastases were presented in Table 3.

**Table 3:** The metastasis presence of included studies

Author (year)	miRNA	Expression level	Clinicopathological details	
			Presence of metastasis	p-value
Du (2017)	miR-543	Low	2	0.005
		High	12	
Fu (2016)	miR-132	Low	7	0.043
		High	6	
Fu (2016)	miR-212	Low	1	0.001
		High	12	
Huang (2017)	miR-141-3p	Low	36	< 0.001
		High	16	
Jiang (2016)	miR-503	Low	9	0.04
		High	2	
Nam (2016)	miR-301a	Low	7	0.5260
		High	7	
Ren (2017)	miR-210-3p	Low	20	< 0.001
		High	48	
Wang (2017)	miR-802	Low	15	0.002
		High	9	
Zhang (2018)	miR-410-3p	Low	17	0.001
		High	33	

### Sub-analysis 1. Association between miRNAs and the lymph node metastasis

Out of eight published papers with the metastasis patients data the analyses selected three articles, describing miRNAs expression among prostate cancer patients with lymph node metastasis. The total number of engaged patients was 353 individuals, constituting approximately 43% of all PCa patients with lymph node metastasis. The heterogeneity of this sub-analysis was at low level and the heterogeneity was not statistically significant (*p* = 0.330, I^2^ = 9.71%). The low expression of miR-503 was associated with the presence of lymph node metastasis (RR = 0.22, 95% CI 0.052–0.989, *p* = 0.047), compared to high expression of miR-503 (Fig. 2b). The results of others two of three miRNAs were not statistically significant (miR-802 and miR-301a).

### Sub-analysis 2. Association between miRNAs and the distant metastasis

From the total eight articles, describing data of metastasis presence, we distinguished four articles, including five miRNAs related to the distant metastasis. This investigated group consisted of 477 individuals, constituting 51% of all patients with metastasis. The heterogeneity analysis for these five miRNAs was at high level (*p* < 0.000, I^2^ = 91.59%). The overexpression of miR-212 and miR-210-3p were found to associate with the higher risk of distant metastasis (RR = 11.59, 95% CI 1.61–83.31, *p* = 0.015; RR = 2.43, 95% CI 1.61–3.67, *p* = 0.000, respectively), compared to loss of miR-212 and miR-210-3p expression. (Fig. 2c). On the other hand, the downregulation of miR-132, miR-802, miR-141-3p were associated with higher distant metastasis risk (RR = 0.36, 95% CI 0.144–0.924, *p* = 0.034; RR = 0.35, 95% CI 0.179–0.692, *p* = 0.002; RR = 0.45, 95% CI 0.277–0.734, *p* = 0.001) in comparison to the up regulated miR-132, miR-802 and miR-141-3p (Fig. 2c).

### Sub-analysis 3. Association between miRNAs and the bone metastasis

Out of eight articles analyzing data of patients with metastases, we distinguished two articles analyzing patients with bone metastases and performed a separate sub-analysis. We decided to include these two articles to sub-meta-analysis apart from the rest types of metastasis. The total number of included patients was 290, which constitutes 35% of all metastatic patients. The heterogeneity test was at high level (*p* < 0.000, I^2^ = 96.27%). The results indicated that high miR-210-3p expression level in prostate cancer tissue was significantly associated with the frequent occurrence of bone metastasis (RR = 2.43, 95% CI 1.61–3.67, *p* = 0.000), compared to low miR-210-3p expression. (Fig. 2d). On the other hand low expression level of miR-141-3p among patients with prostate cancer was associated with the higher risk of bone metastasis occurrence (RR = 0.45, 95% CI 0.27–0.73, *p* = 0.001) in comparison to the high miR-141-3p expression (Fig. 2d).

### Association between miRNAs and the Gleason score

Out of ten articles, we included twelve miRNA molecules to analyze the association between each miRNAs expression level and the total Gleason score > 6. The number of patients was 1448 patients. The heterogeneity test was at middle-high level (*p* = 0.0001, I^2^ = 70%). We distinguished five miRNA molecules with RR value statistically significant. We found that elevated expression of miR-410-3p was significantly associated with Gleason score > 6 (RR = 2.19, 95% CI 1.20–4.00, *p* = 0.01), compared to the loss of miR-410-3p expression level (Fig. 2e). On the other hand, we noticed that decreasing expression of the miR-132, miR-212, mir-503, miR-543 was significantly associated with a total Gleason score > 6 (RR = 0.69, 95% CI 0.49–0.95, *p* = 0.02; RR = 0.66, 95% CI 0.48–0.90, *p* = 0.0007; RR = 0.58, 95% CI 0.39–0.81, *p* = 0.009; RR = 2.14, 95% CI 1.10–4.16, *p* = 0.02), compared to the higher miR-132, miR-212, miR-503, miR-543 expression level (Fig. 2e).

Among eleven publications, we found 13 miRNA molecules, which were included in the analysis of association with the Gleason score > 7. The number of patients was 1630 individuals. The heterogeneity test was middle-high level (*p* = 0.00007, I^2^ = 69.68%). The five of mentioned miRNA molecules were found statistically significant. The analysis showed that low expression level of the miR-212, miR-802, miR-203, miR-543 and miR-573 was significantly associated with the > 7 Gleason score (RR = 0.42, 95% CI 0.21–0.83, *p* = 0.01; RR = 0.38, 95% CI 0.19–0.75, *p* = 0.005; RR = 0.1, 95% CI 0.01–071, *p* = 0.02; RR = 4.33, 95% CI 1.44–13.02, *p* = 0.009; RR = 0.49, 95% CI 0.25–0.96, *p* = 0.04) in comparison to the high miR-212, miR-802, miR-203, miR-543 and miR-573 expression (Fig. 2f). The Gleason score details of studied samples were demonstrated in Table 4.

**Table 4:** The Gleason score presence of included studies

Author (year)	miRNA	Expression level	Clinicopathological details		
			Gleason score		p-value
			≤ 7	> 7	
Bucay (2017)	miR-3622a	Low	89	9	0.0223
		High	22	1	
Colden (2017)	miR-466	Low	57	7	< 0.0001
		High	14	2	
Du (2017)	miR-543	Low	18	3	0.006
		High	8	13	
Fu (2016)	miR-132	Low	14	19	0.028
		High	16	8	
Fu (2016)	miR-212	Low	11	20	0.004
		High	19	7	
Huang (2017)	miR-141-3p	Low	23	48	< 0.001
		High	54	16	
Jiang (2016)	miR-503	Low	11	30	< 0.01
		High	23	17	
Josson (2017)	miR-409-3p	Low			
		High			
Josson (2017)	miR-409-5p	Low			
		High			
Nam (2016)	miR-301a	Low	268	24	0.3634
		High	262	31	
Ren (2017)	miR-210-3p	Low	0	22	< 0.001
		High	0	49	
Saini (2010)	miR-203	Low	10	1	
		High	11	0	
Wang (2015)	miR-573	Low	16	18	0.044
		High	23	8	
Wang (2017)	miR-802	Low			0.004
		High			
Zhang (2018)	miR-410-3p	Low	30 (< 7)	10 (≥ 7)	0.006
		High	19 (< 7)	23 (≥ 7)	

### Association between miRNAs expression level and pT stage

The eleven miRNA molecules from ten publications have been included to analyze of the association of miRNAs expression and pT stage. The patients' number was 1389 individuals. The heterogeneity test was at middle-high level (*p* = 0.0003, I^2^ = 69.53%). The higher risk of the advanced prostate cancer stage was related to the high expression level of miR-410-3p (RR = 2.16, 95% CI 1.23–3.79, p = 0.007) and miR-543 (RR = 3.85, 95% CI 1.52–9.77, *p* = 0.004), compared to the low miR-210-3p and miR-410-3p expression levels (Fig. 2g). On the contrary, the higher risk of the advanced prostate cancer stage was connected to the miR-802 (RR = 0.55, 95% CI 0.34–0.9, *p* = 0.02) and miR-503 (RR = 0.37, 95% CI 0.14–0.92, *p* = 0.03) low expression levels, compared to the high expression level of miR-802 and miR-503 molecules (Fig. 2g). The pathologic tumor stage (pT stage) and details of included samples were shown in Table 5.

**Table 5:** The pathological T stage for tumors of included studies

Author (year)	miRNA	Expression level	Clinicopathological details		
			Pathological/clinical Stage^a^		p-value
			≤ pT2	≥ pT3	
Bucay (2017)	miR-3622a	Low	68	22	1.000
		High	16	1	
Colden (2017)	miR-466	Low	40	3	< 0.0001
		High	13	22	
Du (2017)	miR-543	Low	18^a^	4^a^	< 0.001
		High	6^a^	14^a^	
Fu (2016)	miR-132	Low	13	4	1.000
		High	31	9	
Fu (2016)	miR-212	Low	23	3	0.063
		High	21	10	
Jiang (2016)	miR-503	Low	27	14	0.03
		High	35	5	
Nam (2016)	miR-301a	Low	189	103	0.2968
		High	175	118	
Saini (2010)	miR-203	Low	9	2	
		High	10	1	
Wang (2015)	miR-573	Low	30^a^	11^a^	0.003
		High	18^a^	23^a^	
Wang (2017)	miR-802	Low	10	17	0.02
		High	30	16	
Zhang (2018)	miR-410-3p	Low	29^a^	11^a^	0.003
		High	17^a^	23^a^	

^a^clinical tumor stage

### Regulatory pathways for miRNA-related to EMT program in prostate cancer

**Fig. 3:**
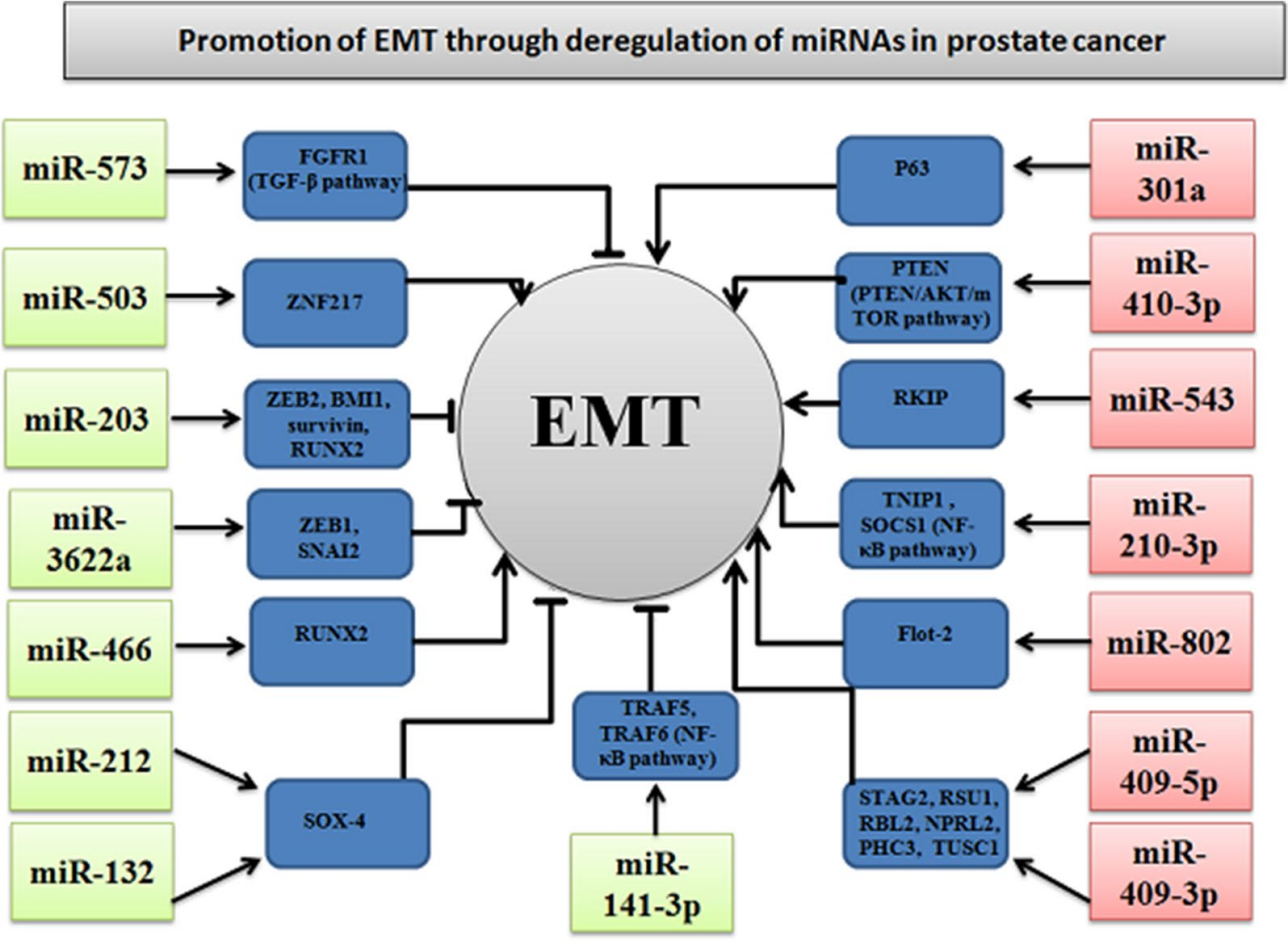
Targets of specific miRNAs-related to EMT program in prostate cancer

**Fig. 4:**
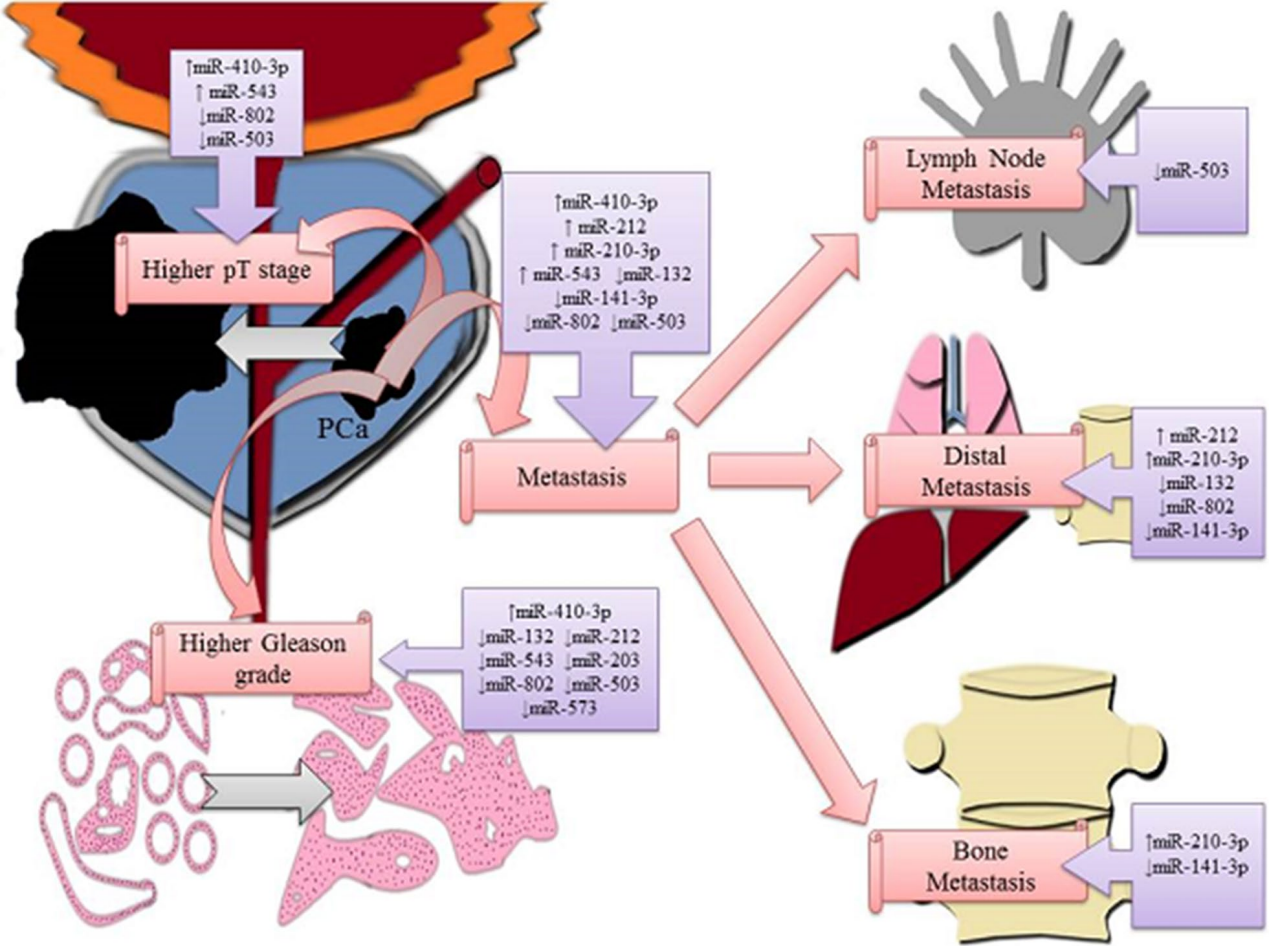
MicroRNA expression signatures related to tumor stage, Gleason grade and progression to metastatic prostate cancer

**Fig. 5:**
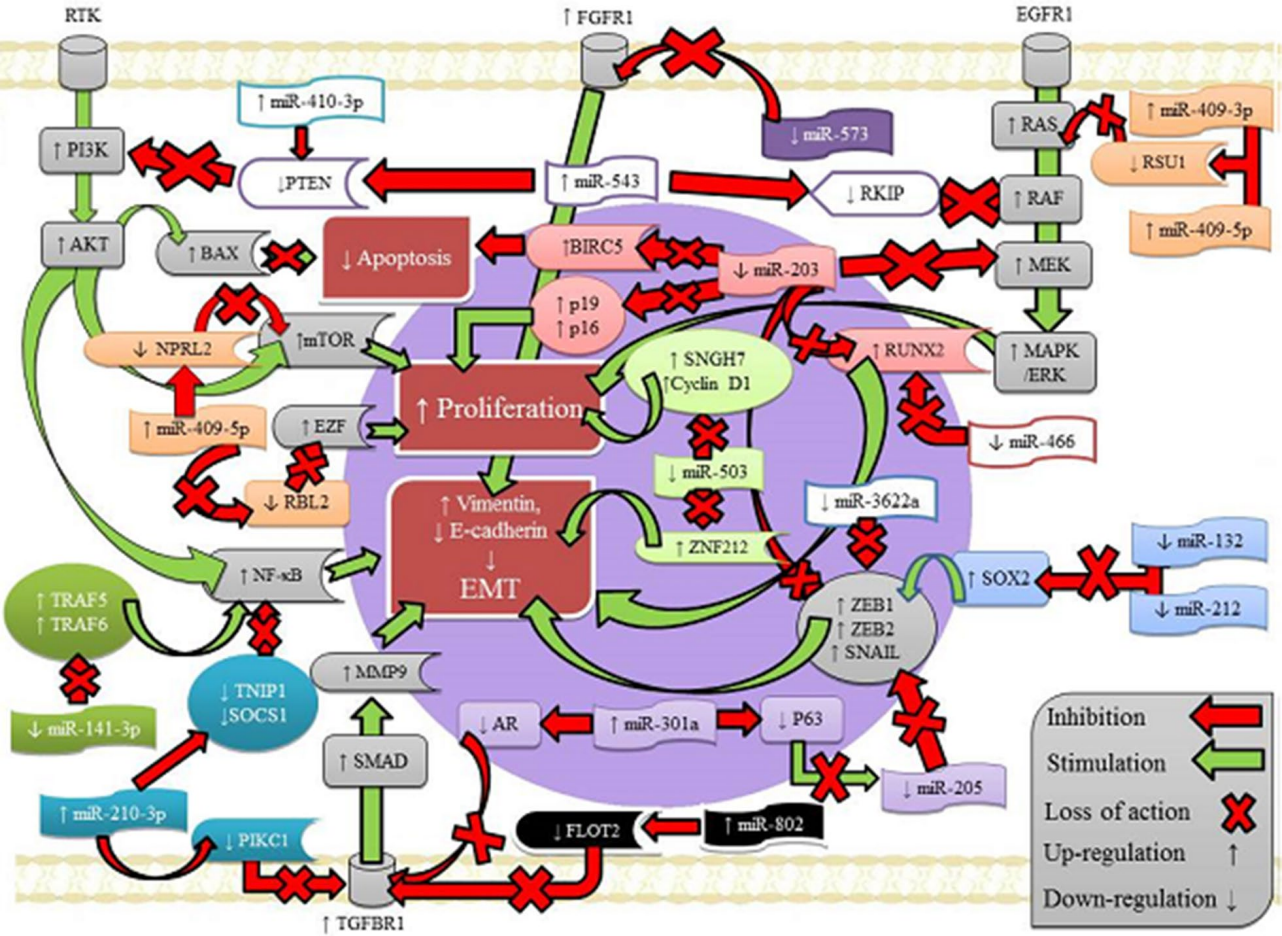
Signaling pathways scheme depicting prostate cancer progression-related targets of dysregulated miRNAs in tumor cells

In the current meta-analysis, we obtained the important results for 15 miRNAs, based on primary data from 13 published studies. For 7 miRNAs (miR-410-3p, miR-301a, miR-802, miR-543, miR-409-3p, miR-409-5p, miR-210-3p) of 13 articles (54%), the deregulation was expressed as an overexpression in prostate cancer samples, while for 8 (62%) of 13 published papers the expression was down regulated (miR-573, miR-3622a, miR-466, miR-503, miR-203, miR-141-3p, miR-212, miR-132). The direct targets of specific miRNAs and their expression alterations in a prostate cancer were presented in the Fig. 3. The associations between specific deregulation of miRNAs expression and following parameters: clinicopathological features, including general, lymph node, distant or bone metastasis, the both Gleason scores > 6 and > 7 as well as pT stage were demonstrated in Table 6. For better understanding aforementioned dependencies, the expression signatures for individual clinicopathological features were demonstrated in Fig. 4. The various mRNA targets of dysregulated miRNAs and their signal transduction pathways in prostate tumor cells were demonstrated in Fig. 5. The presentation of key regulatory molecular mechanisms of analyzed miRNAs involved in progression to metastatic disease supports the understanding of the underlying miRNA functions in prostate cancer cells.

**Table 6:** Association between miRNAs expression and clinicopathological features

	GM	LNM	DM	BM	GS > 6	GS > 7	pT stage
miR-132	↓		↓		↓		
miR-141p	↓		↓	↓			
miR-503	↓	↓			↓		↓
miR-212					↓	↓	
miR-573						↓	
miR-210-3p	↑		↑	↑			
miR-410-3p	↑				↑		↑
miR-543	↑						↑
miR-203						↓	

*GM* general metastasis, *LNM* lymph node metastasis, *BM* bone metastasis, *GS* > *6* Gleason score > 6, *GS* > *7* Gleason score > 7, *pT stage* pT stage according to the TNM system, *↑* overexpressed miRNA, *↓* downregulated miRNA

Many previous studies have indicated that miRNAs play a crucial role in various biological processes, encompassing development, proliferation, differentiation, cell fate determination, apoptosis, angiogenesis, adhesion, metastasis, signal transduction, organ development, hematopoietic lineage differentiation, host-viral interaction and tumor genesis [34–40]. The known biological functions of analyzed miRNAs in the cancerous tissues have been presented in Table 7.

**Table 7:** Biological functions of discussed miRs in cancer

Name of microRNA	Genomic locations	Biological functions in cancer	Ref.
miR-3622a	8p21.1	miR-3622 regulates cancer cells proliferation, progression, apoptosis, G0/G1 cell cycle arrest, migration and invasion abilities of cancer, EMT, metastasis	[23, 41–44]
miR-466	3p23	miR-466 induces proliferation, migration, invasion, angiogenesis cell cycle arrest, apoptosis in cancer cells, EMT and metastasis	[25, 45, 46]
miR-543	14q32.31	mir-543 mediates cell proliferation and tumor growth, apoptosis, the cell cycle, migration, invasion, EMT and metastasis	[30, 47–53]
miR-132	17p13.3	miR-132 regulates proliferation of cancer cells, colony formation, tumor growth, migration and invasion of cancer cells, the G1/S phase transition of the cell cycle, EMT in cancer cells, apoptosis in cancer cells	[54–58]
miR-212	17p13.3	miR-212 mediates the proliferation and invasion of cancer cells, EMT and metastasis	[59–62]
miR-141-3p	12p13.31	miR-141 regulates proliferation, tumor growth, migration, EMT, chemosensitivity, apoptosis, angiogenesis, oxidative stress response, cellular motility and control “stemness” miR-141–3p regulates proliferation, tumor growth, migration, and invasion of cancer cells, cancer cell progression, EMT and metastasis	[33, 63–70]
miR-503	Xq26.3	miR-503 regulates cell proliferation, tumor angiogenesis, cell growth and EMT in cancer cells	[71–77]
miR-409-3p	14q32.31	miR-409-3p mediates tumorigenesis, tumor growth, invasion, EMT and metastasis of tumor cells	[32, 78–81]
miR-409-5p	14q32.31	miR-409-5p mediates tumorigenesis, EMT and bone metastasis	[32, 81]
miR-301a	17q22	miRNA-301a regulates tumor cell invasion, migration, cell proliferation, apoptosis and enhancing chemosensitivity, EMT and metastasis	[82–84]
miR-210-3p	11p15.5	miR-210-3p regulates cell survival, stem cell differentiation, angiogenesis, DNA damage repair, mitochondrial metabolism, immune response, EMT and metastasis	[28, 63, 85–89]
miR-203	14q32.33	miR-203 regulates tumor proliferation, invasion, migration, apoptosis, EMT and metastasis	[90–93]
miR-573	4p15.2	miR-573 mediates cell proliferation, tumor growth, apoptosis, angiogenesis, EMT and invasion of cancer cells	[94–96]
miR-802	21q22.12	miR-802 modulates proliferation, tumor growth, apoptosis, migration, invasion, EMT and metastasis of cancer cells	[29, 97–100]
miR-410-3p	14q32.31	miR-410-3p mediates cell proliferation, invasion, migration, EMT, apoptosis, chemosensitivity and autophagy	[21, 101–103]

### Publication bias

The high heterogeneity level of data of assembled miRNAs could be related to the variables selected in each study. The most frequently heterogeneity sources of variance in meta-analyses are different research methodologies, various patient races, various research samples and methods of sample collection or storage, insufficient data, the small study groups, different analysis periods (follow-up time), various indicators and methods of estimating results. In order to evaluate disturbing factors we performed sensitivity analysis.

**Fig. 6:**
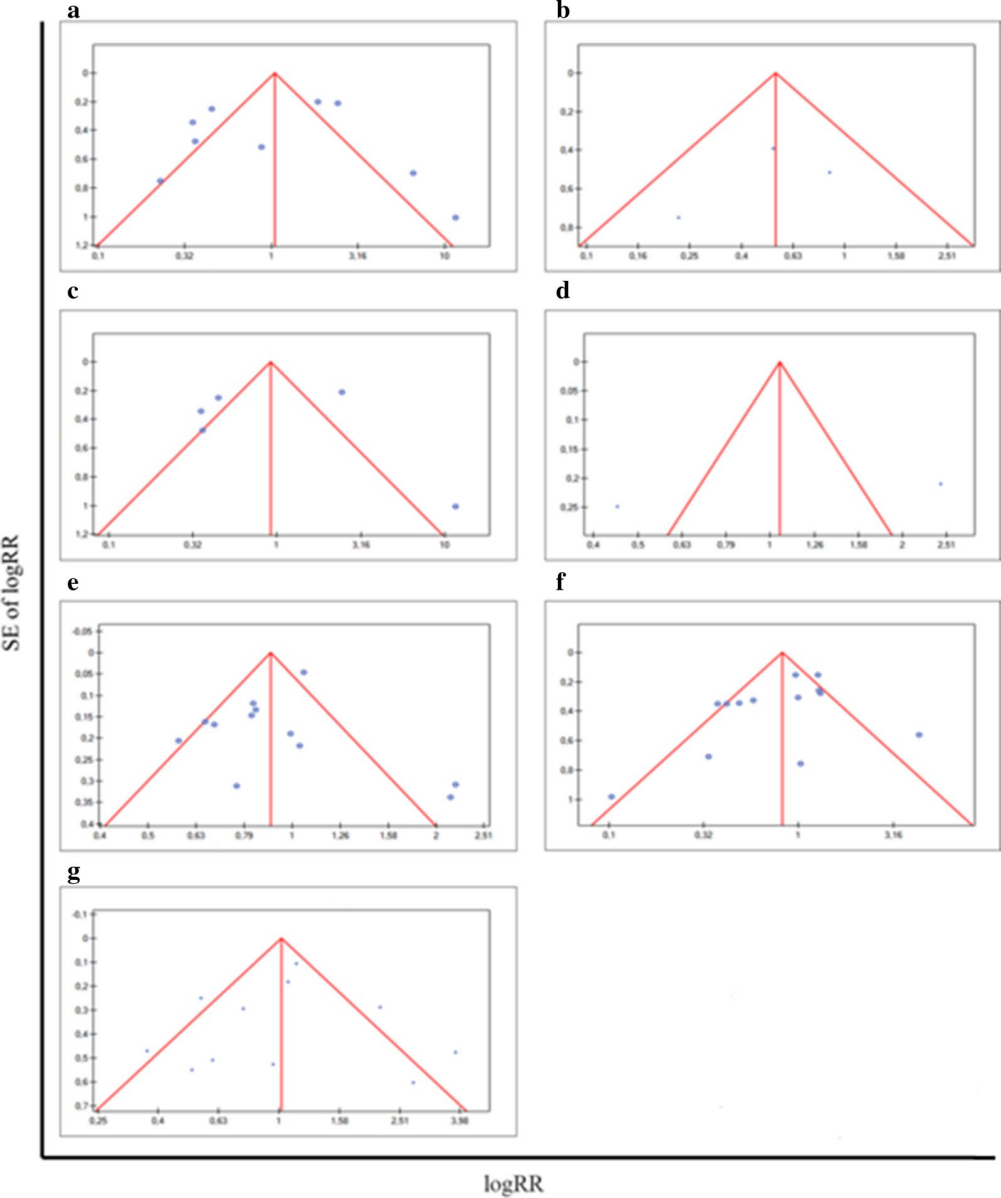
Funnel plots for publication bias

We performed Begg’s funnel plot and Egger’s test to judge potential publication bias of completed analysis. Significant heterogeneities (*p* < 0.05, I^2^ > 50%) were found in a miRNAs and metastasis analysis. The heterogeneity test performed for the analysis of the association of miRNAs and the general metastasis indicated high level (I^2^ = 87.99%) and was statistically significant (*p* < 0.00002). I^2^ > 75% indicates a high heterogeneity of the study, which could be disturbed by different types of metastasis. The main results for pooled RRs for meta-analysis were recapitulated in Table 8. It was right to consider a more homogenous group to meta-analysis in the way of excluding miRNAs, which lead to perturb a heterogeneity of study. In order to reduce the risk of heterogeneity and better presenting of the results, we decided to repeat the analysis formed subgroups based on metastasis type: lymph node metastasis, distant metastasis and bone metastasis. The associations of miRNAs and both presence of distant metastasis and bone metastasis revealed the high level of heterogeneity test and the results was statistically significant (I^2^ = 91.59%, *p* < 0.000001; I^2^ = 96.27%, *p* < 0.000001, respectively). However, the association between miRNAs expression and lymph node metastasis noticed a low level of heterogeneity test at I^2^ = 9.71% and was statistically insignificant. Others compilations indicated a moderate heterogeneity level: I^2^ = 70.09%, *p* = 0.000125 for GS.6; I^2^ = 69.68%, *p* = 0.000085 for GS > 7; I^2^ = 69.53%, *p* = 0.000293 for pT stage. The Egger’s test, counting the asymmetry of conducted analysis, showed statistically non-significant results for all compilations. The funnel plots presenting publication bias were shown in Fig. 6.

**Table 8:** Main results of pooled RRs in the meta-analysis

Comparisions (miRNA groups)	Heterogeneity test			Summary RR (95% CI)	Asymmetrical distribution		Studies
	Q	p-value	I^2^ (%)		b Egger coefficient	p-value	
General metastasis	66.60	< 0.000001	87.99	1.05 (0.52, 2.09)	− 0.86	ns (0.70)	9
Distant metastasis	47.58	< 0.000001	91.59	0.92 (0.32, 2.61)	− 0.82	ns (0.86)	5
Lymph node metastasis	2.21	ns (0.33)	9.71	0.54 (0.30, 1.00)	− 1.80	ns (0.65)	3
Bone metastasis	26.83	< 0.000001	96.27	1.05 (0.84, 5.49)	NA	NA	2
Gleason score > 6	36.78	0.000125	70.09	0.90 (0.77, 1.06)	− 0.71	ns (0.46)	12
Gleason score > 7	39.58	0.000085	69.68	0.82 (0.60, 1.13)	− 1.55	ns (0.15)	13
pT stage	32.81	0.000293	69.53	1.02 (0.73, 1.41)	− 0.31	ns (0.77)	11

### Sensitivity analysis

**Fig. 7:**
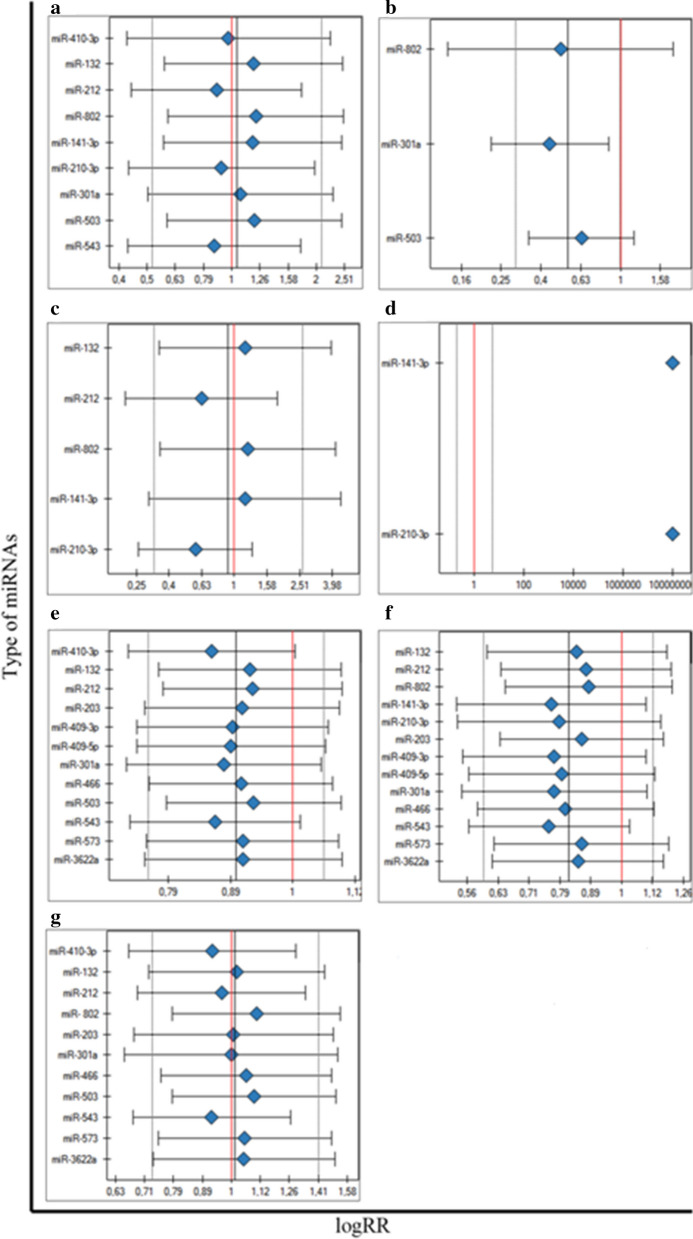
Sensitivity analysis for meta-analysis groups and subgroups

We performed sensitivity analysis to establish the impact of single miRNA studies on the stability of achieved results through removing individual miRNA study, in a sequential manner. For the analysis of association between miRNAs expression level and the general metastasis, we noticed that three studies with miR-212, miR-210-3p and miR-543 had the largest effect on the results, but each *p-*value was statistically non-significant. The rest of the studies showed the RR value close to one or greater than one and constituted stable and robust results. For the comprehensive analysis of individual miRNAs expression and specific metastasis types, the sensitivity analysis demonstrated disorder results for miR-301a, concerning the lymph node metastasis, with the statistically significant *p*-value (*p* = 0.018181) as well as miR-212 and miR-210-3p, concerning the distant metastasis, but the *p*-value was not statistically significant. The RR values of the rest engaged studies of mentioned two analyses were close to each other. For GS > 6, the sensitivity analysis showed two studies with miR-410-3p and miR-543, disturbing the association analysis, but the *p*-value indicated statistically non-significant results. The rest of RR value of miRNA studies was close to one and stable. Similarly, for pT stage, the sensitivity analysis showed three studies (with miR-410-3p, miR-212, miR-543) with RR value, deviating from the rest studies, but the *p*-value revealed statistically non-significant results. The diagrams illustrating sensitivity analysis were included in Fig. 7.

## Discussion

MicroRNAs are molecules which show promise as potential markers of poor prognosis among patients with malignancies. The latest advances in prostate cancer research showed diagnostic, prognostic and treatment utility of variety miRNA molecules measured in serum, plasma, ejaculate, tissue or urine [104–108]. Nonetheless, our study describes the significance of miRNAs involved in the EMT program for the first time, incorporating 1608 prostate cancer patients.

Cancer metastases are a leading cause of cancer mortality and accounts for approximately 90% of deaths due to cancer [109]. However, appropriate diagnostic techniques for predicting progression potential have still not been discovered. Our results provided by 13 tissue-based studies for 7 overexpressed and 8 down regulated miRNAs revealed 9 miRNAs with the highest association with metastatic stage of PCa. The current meta-analysis suggested that miR-210-3p and miR-141-3p had particularly notable significance in a formation of metastasis. Overexpressed miR-210-3p and down regulated miR-141-3p have been found to be associated with the presence of general metastasis, distant metastasis as well as bone metastasis.

MiR-210-3p has been widely recognized as an oncogene, engaged in tumor progression process. MiR-210-3p was documented as up regulated in many cancers: breast cancer [110], lung cancer [111], pancreatic cancer [112], head and neck cancer [113]. In breast cancer, miR-210-3p was recognized in rapid formation of distant metastases [114–116]. Similarly, miR-210-3p expression was found to be elevated in bone metastatic PCa patients compared to non-metastatic PCa patients. Additionally, it was found that miR-210-3p silencing has significantly attenuated bone metastasis formation in PC-3 cells in vivo [28]. As a consequence, it is likely that through the assessment of miRNA levels, metastasis types could be differentiated. Our meta-analysis revealed characteristic miRNAs signatures for each metastasis type. Based on presented results a downregulation of miR-503 seems to be specific for lymph node metastasis while a upregulation of miR-210-3p as well as a downregulation of miR-132 and miR-141-3p are associated to presence of distant metastasis. The signature of aforementioned miRNAs could be potentially used to estimate a risk of metastasis development at an early stage of PCa followed by a more radical treatment procedure in selected cases. On the other hand, the miR-141-3p has been found acting as a tumor-suppressing miRNA, inhibiting the cancer cells progression via directly targeting of tumor necrosis factor receptor-associated factor 5 (TRAF5) in colorectal cancer tissues and cell lines [117]. Similarly, at PCa study, miR-141-3p has been found down regulated, acting as a tumor suppressor that targets TRAF5 and TRAF6 [33].

In the current meta-analysis it was shown that the formation of metastasis overall has been significantly associated with increased expression of miR-410-3p and miR-543. The recent study of Zhang et al. has shown that the elevated expression of miR-410-3p affects a downregulation of phosphatase and tensin homolog deleted on chromosome ten (PTEN) and results in an activation of AKT/mTOR signaling pathway. They have also shown that miR-410-3p plays a crucial role as an oncogene in PTEN/AKT/mTOR pathway [21]. It is possible that its upregulation could promote a disease progression and a metastatic ability of prostate cancer due to deactivation of PTEN protein, a natural inhibitor of PI3K/AKT/mTOR pathway. Similar mechanism may be associated with increased miR-543 level. In colorectal cancer (CRC), miR-543 has been found acting as an oncogene and promotes tumor progression [47]. Moreover, MiR-543 has been significantly up regulated in metastatic prostate cancer tissues [30]. Sun et al. have revealed that PTEN is a direct target of miR-543 [47]. In turn, PTEN has been considered as a tumor suppressing gene, which is strongly involved in the apoptosis process [118]. In the another study, Liu and co-workers have noticed that rising expression of miR-543 may lead to a downregulation of PTEN and inversely, the inhibition of miR-543 could activate PTEN in CRC cells [51]. In many previous studies have been shown that the loss of PTEN gene can increase the PI3K/AKT signal transduction pathway, leading to the tumor progression and formation of metastases [119, 120].

Furthermore, MiR-543 has been also described as a pivotal modulating factor of tumor growth and metastasis through binding to Raf kinase inhibitor protein (RKIP) in PCa. Previous studies have suggested that RKIP is responsible for inhibition of PCa progression by blocking MAP kinase (MAPK) signaling pathway and activation of NF-ƙB factor through G-protein regulating [121, 122]. Li and colleagues in their study at gastric cancer (GC) have demonstrated that miR-543 enhances ability to cell proliferation and formation of colony, what in consequence lead to a progression to the S phase, by directly targeting histone deacetylase sirtuin 1 (SIRT1) [49]. In the same study, the authors have shown that miR-543 molecule negatively modulates the SIRT1 expression in GC cells. Moreover, themiR-543 was positively associated with such clinicopathological parameters as size of the tumor, clinical grade, TNM stage and metastasis to the lymph nodes in gastric cancer patients [49]. Additionally, Haga and co-workers have presented seven miRNAs: miR-300, miR-382, miR-494, miR-495, miR-539, miR-544 and miR-543 in the imprinted DLK1-DIO3 region, which cooperatively regulates the EMT process, by repressing signal transduction network, including TWIST1, BMI1, ZEB1/2, and miR-200 family miRNAs [123]. The epigenetic silencing of miRNAs cluster that appears through hypermethylation of upstream CpG islands in human ductal carcinomas lead to initiate the EMT process [123].

On the other hand, we observed also in current meta-analysis that microRNA molecules might be closely related to a specific type of metastases. The decreased miR-503 expression could correspond with a formation of metastasis generally and lymph node metastasis specifically, while the decreased expression of the miR-132 seems to be associated with the development of metastasis overall as well as with the presence of distal metastasis. These observations may suggest a possibility of determining a risk of lymph node metastasis or distant metastasis development via changes in miR-503 and miR-132 level. MiR-503 has been found to be involved in formation of a malignant phenotype in a wide spectrum of cancers. Using the RT-qPCR, it has been demonstrated the elevated expression level of miR-503 in esophageal squamous cell carcinoma (ESCC). Also, the overexpression of miR-503 was found in correlation with following clinicopathological features: TNM stage, tumor differentiation and lymph node metastasis. Summarizing this study, the authors revealed that miR-503 represses ESCC progression by induction of autophagy, regulated in the PKA/mTOR signaling pathway [124]. Therefore, a decrease in miR-503 level leads to tumor proliferation and metastasize. In human non‐small cell lung carcinoma (NSCLC), miR-503 was observed as a tumor-suppressive miRNA, which was inversely associated with patient overall survival. In turn, upregulation of either PI3K p85 or IKK-β, direct targets of miR-503, guaranteed fractionally restoring of malignant phenotype of cancer cells [125]. Interestingly, in A549/CDDP non-small cell lung cancer cells in comparison to the parental A549 cells, deregulation of miR-503 expression was found to decrease the resistance on cisplatin of NSCLC cells [126]. These findings may have important significance in case of chemotherapy failure due to cancer drug resistance. However, in current work we point out a possibility of miR-503 implementation to predict progression to the lymph node metastasis stage, specifying their potential between miR-503, miR-802 and 301a.

The presence of lymphovascular metastasis constitutes a formidable foundation for the possibility of further extending metastases and forming the tumor cells foci, located at distant body parts. Hence, in the future perspective, a prediction of metastasis development and a clinical classification of metastasis types via fluctuations in microRNA molecules expression levels is justified. Predicting lymph node metastasis by evaluation of miR-503 expression possess a valuable potential in particular for the possibility to inhibit spreading of tumor cells to distant body parts.

Contrarily, miR-132 can play a dualistic role in cancers [127]. In this meta-analysis, miR-132 was found to be associated with both general metastasis and distant metastasis The results of latest studies may confirm an oncogenic role of miR-132 in cancer. Wong and co-workers have found 24-upregulated miRNAs, including miR-132, in tongue squamous cell carcinoma [128]. Liu et al. have revealed that miR-132 was down-regulated in both hepatocellular cancer (HCC) tissues and HCC cell lines and the same miR-132 was responsible for a suppression of tumor development and blocking of invasiveness. Additionally, miR-132 can directly target PIK3R3 and controls the AKT/mTOR signal transduction pathway in HCC [58]. MiR-132 may act as a tumor suppressing miRNA in non-small lung cancer [56] and ovarian cancer [129]. In PCa the loss of miR-132 was found to be in correlation with poor clinical outcome. MiR-132 deregulation leads to decreased a cell-to-cell adhesion and to cell death inhibition, which is necessary for circulating tumor cells to adhere to foreign organ areas. Formosa et al. have observed that the CpC island hypermethylation seems to be responsible for silencing of miR-132 [130].

Such highly heterogeneous tumor as PCa creates significant diagnostic challenges and hinders the ability to predict disease progression. Exploiting the expression alterations of miRNAs specific for kind of metastasis may provide opportunity to early prediction of prostate cancer progression. In this meta-analysis, we want to point out the potential of panel with 6 miRNAs for monitoring disease progression, directed to metastasis occurrence. Our findings revealed that the increased expression of miR-410-3p, miR-210-3p, miR-543 and the decreased expression of miR-132, miR-141-3p and miR-503 have been significantly associated with the appearance of metastases in progress of PCa.

The second aim of our study was to analyze a relationship between miRNAs in PCa and a Gleason score. The Gleason scoring system constitutes an especially important prognostic factor associated with the growing aggressiveness of neoplastic changes in the prostate. The increased Gleason grade has been connected inter alia with pathologic stage, tumor size, margin status, progression to metastatic form and patient survival [131]. Total Gleason score is one of the main factors involved in making treatment decisions, together with pathological stage and pre-PSA level [132].

In our meta-analysis we decided to compare the miRNAs importance in the set of “Gleason score above 6” and in the set of “Gleason score above 7”. Aberrant miR-410-3p, miR-132, miR-212, miR-503 are separately the most associated with total Gleason score > 6 and abnormal expression of miR-212, miR-203 and miR-573 are individually the most associated with Gleason score > 7 in PCa tissue. MiRNAs molecules most integrated with both Gleason scores above 6 and 7 is miR-212. The future implementation of diagnostic tests to capture the variations in miRNAs expression among prostate cancer patients limiting to Gleason score, suggests the opportunity of monitoring the patient’s condition and disease progression at early cancer stage, excluding need to resection of prostate gland or radiotherapy.

The miR-212 was found to be upregulated in pancreatic ductal adenocarcinoma in comparison to the health tissue. In the current study, it has been shown the positive correlation between deregulation of miR-212 and TNM stage, lymph node metastasis, vessel invasion, size of the tumor and the overall survival time. Latest, it has been found that in hypoxic conditions miR-212 aberration was correlated with the hypoxia-inducible factor (HIF-1α) in vivo and in vitro [133]. In turn, in cervical cancer tissues and cell lines, miR-212 was found outstandingly down regulated and promoted proliferation and invasion of cancer cells. Using the Western blot tool the authors have indicated that miR-212 upregulation notably blocked the transcription factor 7-like 2 (TCF7L2) protein expression level [134].

Furthermore, miR-212 contributes in the miR-212/132 cluster in human lung cancer [135]. Interestingly, the current study also showed an association between miR-132 level and Gleason score > 6. Jiang et al. have suggested that overexpression of miR-212/132 cluster remarkably blocks the growth and invasiveness of tumor cells in A549 and H1299 lung cancer cell lines [135]. Additionally, miR-212/132 upregulation leads to activation of cell cycle arrest at the G1/S phase transition via regulation of p21 and cyclin D1 expression [135]. Upregulation of miR-132/212 has been found in drug resistant breast tumors and cell lines. Moreover, the miR-132/212 has been shown responsible for regulation of drug accumulation through blocking the PTEN expression levels in vitro. Also, downregulation of PTEN has been found inversely associated with miR-132/212 expression in drug resistance breast cancers. Upregulation of miR-132/212 cluster was partially connected with transactivation via the NF-ƙB transcription factor and this mechanism may have importance in acquisition of drug resistance [136]. Additionally, current statistical analysis showed that another aforementioned miRNAs, miR-410-3p and miR-503 was found to be in association with Gleason score value > 6, which demonstrates their potential prognostic role at an early stage of prostate cancer development.

MiR-573 and miR-203 was found to be associated only with Gleason score > 7, therefore we consider that these molecules exhibit a lower prognostic value for predicting EMT. However, miR-573 and miR-203 can be used to predict Gleason score ranges from 7 to above. Wang et al. have investigated that upregulation of miR-573 may be stabilized by miR-573 mimic, leading to inhibition of proliferation and progression in melanoma cells [137]. On the other hand, Wang and other authors have revealed that downregulation of miR-573 in PCa is precisely associated with the Gleason score and cancer-related mortality (p = 0.41). The latest study showed that miR-573 could regulate the activity and function of Fibroblast growth factor receptor 1 (FGFR1) in response to fibroblast growth factor 2 (FGF2). Additionally, in the same study, transcription factor GATA3 was indicated as an enhancer of miR-573 expression, causing downregulation of FGFR1, EMT and invasion of PCa cells [22].

MiR-203 has been found to be a tumor suppressing miRNA in some cancers: hepatocellular cancer [138], breast cancer [139, 140], esophageal cancer [141], leukemia [142], glioma [143], bladder cancer [144], leading to inhibition EMT by targeting various genes and downregulation of signal transduction pathways, thus is involved in cancer progression (tumor proliferation and invasion). However, its role in some cancers has been found different. MiR-203 can function as an oncogene in ovarian cancer [145]. In SKOV3 and OVAR3 ovarian cancer cell lines, high expression of miR-203 leads to restrain of EMT process by linking to BIRC5 and causes its downregulation and attenuates the TGF-β signal transduction pathway [92, 146]. Chen and Ding et al. have investigated the effect of elevated miRNA expression on PC-3M prostate cancer tumor cells behavior and they found that downregulation of miR-203 was connected with adriamycin (ADM) resistance, thus elevation of miR-203 repress tumor cells proliferation, enhance apoptosis and reduce ADM resistance through influencing on MAPK kinase 1 (MEK1) expression [147]. MEK1 is responsible for phosphorylation of the Tyr/Thr residue on ERK protein and leads to induction of ERK/MAPK transduction pathway. In turn, excessive stimulation of ERK/MAPK pathway intensifies progression of tumor cells [147].

Finally, we analyzed the association between miRNAs and the pT stage of PCa. The process of determining tumor stages among prostate cancer patients relies on evaluation of probability of tumor spread before including the individual treatment [148]. The increased expression of both miR-410-3p and miR-543 as the same as decreased expression of miR-503 have a significant relationship with rising probability of tumor extending and higher T stage at the time of diagnosis. Our results may be useful for prediction of prostate cancer progression at an early stage. Forasmuch as further research is needed in the prostate cancer progression area, it is important to be prudent in devising far-reaching conclusions. However, it was noticed that progression to a higher pT stage is associated with the aberration of 3 miRNA levels: miR-410-3p, miR-543 and miR-503 involved in invading surrounding tissues and metastasizing. Du et al. at their study on CRC, using gene chip technology grouped inter alia by stage, have proven that microRNA expression profiles were significantly different at various CRC stages, thereby confirming that miRNAs are engaged in a cancer development and in particularly are significant at early diagnosis or cancer prognosis [149]. Concluding, the probability of cancer progression to its metastatic stage can be assessed at an early stage of tumor development. MiR-410-3p play a different role in various cancers. Zhang et al. have noticed that increased expression of miR-410-3p repress proliferation and invasion process in the breast cancer cells [150]. These authors also displayed that transcription factor Snail is a direct target of miR-410-3p and in this way miR-410-3-p inhibits an EMT process. MiR-410-3p has been demonstrated to inhibit the invasion and migration of rhabdomyosarcoma cells through inhibiting EMT [151]. Wang and other authors suggested that miR-410 function in CRC may be connected to negative regulation of DKK-1 through Wnt/β-catenin pathway [152]. MiR-410-3p plays a significant role as an oncogene in PCa via PTEN/PI3K/AKT pathway. Downregulation of miR-410-3p leads to an activation of PTEN, which is a crucial modulator of the PI3K/AKT signaling pathway [21]. Similarly, the role of miR-543 in tumor progression and metastasis were confirmed in many earlier publications [52, 153, 154]. It has been also reported that miR-543 shows outstanding overexpression in the metastatic PCa cell lines compared to the normal cells. Additionally, Raf kinase inhibitor protein (RKIP) was found to be a direct target of miR-543. Upregulation of miR-543 leads to decrease in expression level of RKIP and promotes proliferation and metastasis in PCa cells. [30]. Previous studies have revealed that described molecules are undeniably involved in initiation EMT transition, therefore they are ideal candidates for metastasis risk prediction.

### Diagnostic, prognostic and therapeutic aspect of analyzed miRNAs

The revolution in the diagnosis of prostate diseases was measuring PSA level in blood samples, however it is questionable due to the lack of association between PSA level and prostate cancer stage, which may result in overdiagnosis and overtreatment. Currently, many scholars put immense effort into identifying new biomarkers to improve the diagnosis and prognosis accuracy of PCa entities. Previous studies have indicated that specific miRNA profiles were able to distinguish cancerous and non-cancerous tissues [155], incoming the potentiality of miRNAs in early cancer detection, prognosis and monitoring of treatment response.

Tissue biopsies represent a gold standard in prostate cancer patients’ diagnosis. Nevertheless, tissue sampling technique is susceptible to misdiagnosis, whereas a negative outcome cannot completely exclude presence of cancer foci. Hence, the researchers are looking for a better diagnostic tool. miRNA molecules have multiple advantages in diagnostic or treatment approach, for example, RNA has a higher specificity and its expression alterations are characteristic for individual disease. The expression of miRNA in cancer cells is a process during which can be observed changes that appear over disease development. Additionally, in comparison of tissue-based miRNAs with detection of miRs in bodily fluids, the second one mentioned can be associated with many factors, such as age, diet or circadian rhythms [156]. miRs are less subjected to degradation due to their small size. Additionally, miRs possess great stability during FFPE tissue processing since they are encapsulated in exosomes [156].

Current research state constitutes that non-coding RNA, such as miRNAs, has great potential for PCa diagnosis. An individual gene can be targeted by various miRNAs, which accelerate atypical cell growth and provoke cell death [157]. Furthermore, miRNAs can manage cancer-related processes, such as EMT or metastasis. MiRNAs can be measured both by tissue-based biopsy or in various bodily fluids [158]. Aghdam and colleagues indicated that tissue miRNAs are appropriate for diagnosis and staging of malignancies as well as minimize the necessity for numerous biopsies to determine the appropriate diagnosis [158]. Even if the miRNA detection on tissue-based biopsies shows multiple benefits for prostate cancer patients, it is not yet applied in present clinical practice.

Certain miRNA molecules described in current meta-analysis have been studied previously for diagnostic and prognostic potential in prostate cancer patients. MiR-141 in association with miR-151-3p and miR-16 was found to have a sensitivity of 84% and a specificity of 96% regarding mCRPCa detection from liquid samples [159]. Agaoglu and colleagues presented that circulating miR-141 in compilation with miR-21 and miR-221 have potential to differentiate PCa patients in metastatic stage from those with local advance stage [160]. Other authors have shown that the combination of miR-141-3p with miR-21 and miR-375 presented a sensitivity of 93% and a specificity of 63% during prediction of PCa in serum samples [161]. MiR-141 is a widely researched molecule in various cancers, useful at each stage of diagnosis and prognosis for prostate cancer patients.

Also miR-301a was found to be a valuable diagnostic and prognostic biomarker for prostate cancer disease. The study performed on 28 prostate cancer patients and 13 controls as well as 40 radical prostatectomy cases indicate the possible correlation between miR-301a detected in serum and Gleason score [162].

MicroRNAs are validated as necessary tools in designing biomarker panels for identification prostate malignancies primarily in early but also advanced stages. Regulation of aberrant miRNA expression will repress the targets responsible for progression to higher prostate stage. Presently, TNM staging along with Gleason score and pretreatment PSA levels are utilized to predict the outcome for prostate cancer patients. The microRNA analytes in current meta-analysis have potential for higher accurate prostate cancer staging and developing therapy schemes. Additional advantage for miRNA diagnostic value in prostate cancer is the fact that miRs are situated at fragile chromosomal sites corresponding with tumor hotspots [12].

The role of miRNAs in prostate malignancies is not only limited to diagnosis or prediction and prognosis. MiRNA molecules can also function as targets or therapeutic agents. MiRNA molecules can regulate multiple target genes, therefore alterated expression constitutes a potential therapeutic value for the balance of gene expression [163]. Aside from direct application of miRNA as a valuable therapeutic option, miRNAs can facilitate selection of the best individual treatment scheme and predict response to a personal therapy [158]. The individual patients' response to a treatment application, such as radical prostatectomy, radiotherapy, testosterone suppression and hormone therapy, chemotherapy and immunotherapy is different [158, 164]. Intriguingly, miRNA can be applied as adjuvants acting as an enhancer of tumor sensitivity to therapy [158].

Zedan and other authors have investigated the relationship between circulating miR-141 and miR-375 and treatment outcome in metastatic castration resistant prostate cancer (mCRCP), where 40 patients were treated with docetaxel and 44 with abiraterone [165]. The results of the mentioned study showed prognostic importance of miR-141 and miR-375 in treatment response monitoring among mCRPC patients. Intriguingly, Gonzales and colleagues noticed a strong correlation between miR-141 levels and chemotherapy response in patients with confirmed metastatic stage of prostate cancer. Furthermore, they proved that multivariate panel of directional alterations in PSA, circulating tumor cells (CTCs) and miR-141 had higher sensitivity of 78.9% in clinical outcome prediction [166]. Saini and other authors have studied miR-203 in advanced metastatic prostate cancer and revealed that miR-203 may act as an “antimetastatic” miRNA at numerous steps in the metastatic process by repression of prometastatic targets. Hence, miR-203 has been recognized as an attractive target for patient therapy in advanced prostate disease [27]. The prognostic value of miR-141 and miR-409-3p was demonstrated by Nguyen and other authors, investigating the differential expression of 669 miRNA prostate cancer serum. They confirmed that miR-141, miR-375 and miR-378 increase-depend manner with progression to higher prostate cancer stage, while miR-409-3p was marked to be overexpressed in high-risk group compared to low-risk group, but significantly reduced in the metastatic CRPC, which probably was associated with the androgen deprivation therapy [167]. Watahiki and colleagues proposed miRNA panel, including miR-141, miR-152 and miR-423-3p significantly deregulated in metastatic CRPC compared to localized PCa emerged from 742 miRNAs studied in plasma samples, demonstrating AUC of 0.944. Additionally, the researchers confirmed that miR-141 together with miR-151-3p, miR-152 and miR-423-3p was linked with poor outcome and higher Gleason score. Furthermore, miR-141 and miR-152 was able to detect a high probability of recurrence among patients after radical prostatectomy [159]. In contrast to serum-based miRNA studies, Porkka et al. have profiled 319 miRNA molecules in nine PCa and compared to them four BPH tissues, shown inter alia miR-210 together with other 7 miRNAs up regulated and 22 miRNAs down regulated [168]. Interestingly, Srivastava et al. have shown 30 miRNAs, including miR-210 and miR-212 down regulated and three up regulated in 40 FFPE tissue specimen blocks, comparing PCa tissues to adjacent normal tissue [169].

Clinical relevance of miRNA molecules constitutes the basis to widespread research in the therapeutic context. Therefore, investigators are underway to explore systems balancing miRNA expression levels, onco-miRNA antagonizing and TS-miRNA replacement systems. Modified anti-miRNA systems (microRNA mimetics–miRNA-mimic) can imitate or inhibit cellular miRNA functions to silence complex signal transduction pathways and control multiple physiologic functions [170]. Currently, miRNAs-based therapeutic approach constitutes a high-potential weapon to treat such heterogeneous disease, in terms of molecular abnormalities, pathologic tumor growth patterns and patients outcomes [171] as prostate cancer.

Currently, miRNAs are a part of multiple clinical trials exploring genomic signatures to solve a problem with patients’ selection and recognize companion biomarkers for conventional therapeutic approach. One of the prostate cancer clinical trial is interventional study for 50 patients with low-risk prostate cancer [tumor stage cT1-2a cN0 cM0 and Gleason Score of index lesion ≤ 6 (3 + 3)] to investigate the feasibility and toxicity of focal brachytherapy. A secondary outcome for this phase II clinical trial was to verify the correlation level of miR-141 and miR-375 expressions with visible efficacy of radiation intervention (ClinicalTrials.gov Identifier: NCT02391051). Previously, miR-141 together with miR-21 and miR-221 have been studied in the plasma of 51 prostate cancer patients with locally advanced and metastatic prostate cancer, where miR-21 was related to malignancy state and miR-141 was found to be capable of distinction localized and metastatic stage of prostate cancer [160]. Another one pre-clinical study of 667 samples of serum obtained from prostate cancer patients recognized miR-141 and miR-375 as molecules strongly involved in disease progression and were considered as non-invasive blood-based circulating biomarkers for prostate cancer progression [172]. More precisely, Brase and colleagues noticed that overexpression of miR-141, miR-200b and miR-375 in prostate cancer patients serum was associated with higher tumor stage and Gleason score [172]. Cheng et al. have analyzed panel of 365 miRNAs in prostate cancer serum and emerged miR-141, miR-200a, miR-200c, miR-375 and miR-210 molecules, which was significantly expressed in metastatic CRPC patients compared to age-matched healthy controls as well as in lymph metastasis samples [173]. In another study, Kelly et al. demonstrated a normalization in miR-141 expression in prostate cancer patients who underwent a radical retropubic-prostatectomy 10 days post-operation [174, 175]. Hao et al. have found miR-141 and miR-21 twofold up regulated, while miR-10, -16, -34c and -125b down regulated in FFPE tissue compared to BPH specimens [176]. Additionally, association of PSA level with miR-141 and miR-21 expressions enhanced the positive predictive value from 40 to 87.5% [177].

Improvement in survival rate for prostate cancer patients follows from the advances in first line treatment approach like surgery, radiation and antagonists to the androgen signaling axis [156]. However, the metastatic stage of prostate cancer has no treatment opportunities due to the resistance to androgen antagonists. In turn, FDA was approved taxane-based therapy to provide patients three months-longer overall survival [178, 179]. Another interventional study concern 211 prostate cancer patients at newly diagnosed metastatic stage (stage IV) (ClinicalTrials.gov Identifier: NCT01120236), were as a secondary outcome was measured correlation level between miR-141, miR-210, miR-200b and miR-375 as well as PSA level and circulating tumor cells (CTC). In this phase II randomized trial prostate cancer patients were treated with bicalutamide, goserelin, or leuprolide acetate in combination with cixutumumab or without cixutumumab. Another one observational cohort trial has recruiting status and attempts to identify the role of exosomal microRNA and define microRNA expression profiles to predict the aggressiveness of PCa in urine (ClinicalTrials.gov Identifier: NCT03911999).

## Conclusion

PCa is one of the commonest malignancies in Western countries. The GLOBOCAN STATISTICS 2018 indicates at 7.1% of all new cancer incidence worldwide in 2018 year (for both sexes) [180]. MiRNA molecules offer new possibilities for diagnostic and systemic therapeutic approaches. They can attenuate or obstruct multiple networks involved in the tumor microenvironment, not only single transduction pathway [181]. Furthermore, miRNAs can be a more effective weapon against tumors as they are secreted from malignant cells and their anomalies in expression levels are associated with progression of prostate diseases.

We consider that the higher prognosis value is presented by sub-analysis associating miRNAs expression and GS > 6, showing association with miR-410-3p which was also connected with the presence of general metastasis and the pT stage, miR-503 with significance in general or lymph node metastasis and pT stage and miR-132 connected to general metastasis and distant metastasis. Mentioned miRNAs molecules could be used as a miRNA panel to determine the progression risk at early stages of the disease. Our findings present future potential therapeutic options to treat men with PCa and could provide better post-treatment care. The above mentioned experiments strongly indicated that miRNAs expression modification utilizing miRNA mimics or antagomirs can harmonize gene regulatory circuits and signal transduction pathways. Also, such activities may stop the aggressive status of tumors and reverse the cancerous status of cells.

## Data Availability

All data generated or analyzed during this study are included in this published article.

## Abbreviations

PCa: Prostate cancer
EMT: Epithelial–mesenchymal transition
miRNAs: MicroRNAs
mRNAs: Messenger RNAs
Onc: Oncomirs
FFPE: Formalin-fixed paraffin embedded tissue
TNM: TNM Classification of Malignant Tumor System
GS: Gleason score
HRs: Hazard ratios
RRs: Relative risks
CIs: Confidence intervals
I^2^: Inconsistency index
NOS: Newcastle–Ottawa Scale
RT-PCR: Real time polymerase chain reaction
pT stage: Pathologic tumor stage
TRAF5: Tumor necrosis factor receptor-associated factor 5
CRC: Colorectal cancer
MAPK: MAP kinase
GC: Gastric cancer
HIF-1α: Hypoxia-inducible factor
PTEN: Phosphatase and tensin homolog deleted on chromosome ten
FGF2: Fibroblast growth factor 2
MEK1: MAPK kinase 1
RKIP: Raf kinase inhibitor protein
